# Bevacizumab and Paclitaxel in Advanced, Hormone Receptor-Positive Breast Cancer: Multifactor Dimensionality Reduction Methodology to Identify Best Overall Survival

**DOI:** 10.32604/or.2026.073799

**Published:** 2026-04-22

**Authors:** Luigi Coltelli, Paola Orlandi, Chiara Finale, Gianna Musettini, Luna Chiara Masini, Marco Scalese, Giulia Soria, Elena Sartori, Ylenia Nodari, Giada Arrighi, Arianna Bandini, Marta Banchi, Costanza Tacchi, Donghao Tang, Barbara Salvadori, Lucia Tanganelli, Simona Giovannelli, Mirco Pistelli, Samanta Cupini, Maurizio Lucchesi, Alessandro Cosimi, Giulia Lorenzini, Elisa Biasco, Chiara Caparello, Giulia Acconci, Eloise Fontana, Eleonora Bona, Azzurra Farnesi, Antonio Pellino, Andrea Marini, Ermelinda De Maio, Irene Stasi, Cecilia Barbara, Enrico Sammarco, Javier Rosada, Giacomo Allegrini, Guido Bocci

**Affiliations:** 1 Department of Oncology, Azienda USL Toscana Nord Ovest, Livorno, Italy; 2 Division of Medical Oncology, Pontedera Hospital, Azienda USL Toscana Nord Ovest, Pontedera, Italy; 3 Department of Translational Research and New Technologies in Medicine and Surgery, University of Pisa, Pisa, Italy; 4 Division of Medical Oncology, Livorno Hospital, Azienda USL Toscana Nord Ovest, Livorno, Italy; 5 Institute of Clinical Physiology, Italian National Research Council—CNR, Pisa, Italy; 6 Division of Medical Oncology II, Azienda Ospedaliero-Universitaria Pisana, S. Chiara Hospital, Pisa, Italy; 7 Division of Medical Oncology, Versilia Hospital, Azienda Usl Toscana Nord Ovest, Lido di Camaiore, Italy; 8 Division of Medical Oncology, San Luca Hospital, Azienda Usl Toscana Nord Ovest, Lucca, Italy; 9 Division of Medical Oncology, Umberto I Salesi-Lancisi Hospital, Azienda Ospedaliero-Universitaria Umberto I, Ancona, Italy; 10 Division of Medical Oncology, Apuane Hospital, Azienda Usl Toscana Nord Ovest, Massa e Carrara, Italy; 11 SC Screening, Azienda USL Toscana Sud Est, Siena, Italy; 12 Department of Internal Medicine, Azienda USL Toscana Nord Ovest, Livorno, Italy; 13 Division of Internal Medicine, Livorno Hospital, Azienda USL Toscana Nord Ovest, Livorno, Italy

**Keywords:** Single nucleotide polymorphisms, vascular endothelial growth factor-A, VEGF receptor-2, bevacizumab, paclitaxel, advanced breast cancer, angiogenesis, pharmacogenetic interaction analysis

## Abstract

**Background:**

The treatment of advanced hormone receptor-positive (HR+) breast cancer has seen relevant changes in last years. However, bevacizumab remains an option when combined with paclitaxel, but no certified pharmacogenetic profiles are now usable for the prediction of its response in breast cancer patients. This study aimed to explore the pharmacogenetic interactions among single nucleotide polymorphisms (SNPs) of genes involved in the angiogenic process and their impact on progression-free survival (PFS) and overall survival (OS) in hormone receptor-positive (HR+) metastatic breast cancer subjects administered with bevacizumab plus paclitaxel, or with paclitaxel alone (clinicaltrial.gov identifier NCT01935102).

**Methods:**

Germline DNA extracted from blood samples was analyzed using real-time polymerase chain reaction to investigate SNPs. The multifactor dimensionality reduction (MDR) analysis was employed to assess interactions between these genetic variants. A total of 168 eligible patients were analyzed. Among these, 106 patients received both paclitaxel and bevacizumab, while 62 received paclitaxel alone.

**Results:**

In the combination therapy group, MDR analysis identified two pharmacogenetic interaction profiles involving specific genotypes of vascular endothelial growth factor-A(VEGF-A) rs833061 and vascular endothelial growth factor receptor-2 (VEGFR-2) rs1870377. Patients with a favorable genetic profile had a median PFS (mPFS) of 22.9 months, compared to 8.7 months in those with an unfavorable profile (*p* = 0.001). Cox proportional hazards analysis displayed an adjusted hazard ratio of 0.443 (95% CI: 0.284–0.691; *p* < 0.0001). The median OS (mOS) was 50.2 months for the favorable profile vs. 23.5 months for the unfavorable (*p* = 0.003), with an adjusted hazard ratio (HR) of 0.404 (95% CI: 0.249–0.657; *p* < 0.0001). In the 62 subjects administered with just paclitaxel, no significant differences in PFS (*p* = 0.820) or OS (*p* = 0.143) were observed between favorable and unfavorable genetic profiles.

**Conclusions:**

The MDR analysis of VEGF-A rs833061 and VEGFR-2 rs1870377 genotypes can detect a subgroup of bevacizumab-administered+ metastatic breast cancer patients with improved PFS and OS.

## 1 Introduction

The treatment of advanced breast cancer patients with hormone receptor-positive (HR+) and HER2-negative (HER2-) tumors has seen relevant changes in last years. Cyclin-Dependent Kinase 4/6 Inhibitors (CDK 4/6i) palbociclib [1], ribociclib [2], and abemaciclib [3], combined with aromatase inhibitors or fulvestrant, have become the standard of care both in the first- and subsequent line of therapies [4,5], with a median overall survival (mOS) in first-line that can overcome 50 months. Recently, the CDK 4/6i effectiveness has also been demonstrated in the adjuvant setting [6–9]. If CDK 4/6i therapies fail, hormonal manipulations are still available, but comparative studies are lacking, making it difficult to define a standard of cure.

Novel endocrine therapies based on phosphoinositide-3-kinase (PI3K)/AKT pathway alterations, estrogen receptor 1 (ESR1) mutations, and poly(ADP-ribose) polymerase inhibitors (PARPi), have a new role to play. ESR1 mutations are often developed during aromatase inhibitor (AI) therapy. The oral selective estrogen receptor degrader (SERD) elacestrant [10] has demonstrated in Emerald trial superior median progression-free survival (mPFS) (2.8 vs. 1.9 months, hazard ratio [HR], 0.70, 95% CI, 0.55 to 0.88) compared to standard endocrine therapy in advanced hormone receptor-positive breast cancer compared to standard endocrine therapy in advanced hormone receptor-positive breast cancer [11]. New oral SERDs, under development, such as imlunestrant [12] plus abemaciclib, have demonstrated, in the EMBER trial, a major mPFS (5.6 vs. 5.5 months, HR, 0.87, 95% CI, 0.72 to 1.04) in the ESR1-mutant population, compared to imlunestrant alone [13]. For patients with PIK3CA mutation, inavolisib [14] combined with palbociclib represents a therapeutic opportunity in the first-line setting due to results from the INAVO 120 trial, which demonstrated superior mPFS compared with fulvestrant and palbociclib (15 vs. 7.3 months, HR, 0.43, 95% CI, 0.32 to 0.59) [15]. Capivasertib [16], an AKT inhibitor, combined with fulvestrant, showed a superior mPFS (7.2 vs. 3.6 months, HR, 0.60, 95% CI, 0.51 to 0.7) vs. fulvestrant alone in the CAPitello291 trial [17]. In addition, PARPi olaparib [18] or talazoparib [19] represent an option in patients with BRCA1 or BRCA2-mutant germline tumors, for the results of OlympiAD, which demonstrated improvement in mPFS (7.0 vs. 4.2 months; HR, 0.58; 95% CI, 0.43 to 0.80), and for the EMBRACA study (8.6 vs. 5.6 months; HR, 0.54; 95% CI, 0.41 to 0.71; *p* < 0.001) compared to standard chemotherapy [20].

Though Akt inhibitors, oral selective estrogen receptor degraders, or PARP inhibitors [11,21–23], can represent an alternative, relapse occurs in the whole population of patients with HR+ metastatic disease, and chemotherapy is deemed necessary. In this scenario, the guidelines generally support monotherapy over combinations in the perspective of a sequential strategy [24–26].

The addition of the monoclonal antibody bevacizumab [27] to paclitaxel [28] improves progression free survival (PFS) compared to chemotherapy alone [29–31]. While the US Food and Drug Administration (FDA) cancelled the first consent of the use of the antiangiogenic antibody in first-line therapy of metastatic breast cancer (MBC) patients due to the absence of clinical advantage in terms of overall survival (OS), the European Medicines Agency (EMA) did not. The rationale why many argue that bevacizumab remains an option when combined with paclitaxel depends on the long survival awaited after the first-line schedules in patients harboring breast cancer, which could apparently impact the lack of OS [29,32]. Different experimental approaches have been explored to detect biomarkers that can predict which patients are most likely to respond to bevacizumab plus chemotherapy. While PFS improvement with bevacizumab was consistent across different patient subgroups [33], new selective biomarkers are needed to identify patients with increased OS [34]. Among new predictive biomarkers, a possible role could be ascribed to MALAT1, a huge non-coding RNA that modulates gene expression, that interacts with many signaling pathways, including the angiogenic ones [35], or to circular RNA [36]. Moreover, the exploration of new mechanistic insights on anticancer drugs with specific biological targets and signaling pathways [37] and the advancement in precision diagnosis and therapeutic approaches [38] may open the way to new biomarkers to predict survival of breast cancer patients.

Despite many previous efforts, no certified biomarkers can be used for clinical routine. Indeed, the MERiDiAN trial did not show any potential capacity of baseline VEGF-A plasma concentration in predicting the efficacy of bevacizumab in breast cancer subjects [39,40].

Germline and somatic polymorphisms in genes involved in the process of angiogenesis were extensively investigated to improve bevacizumab efficacy, but the results have been contradictory [41,42]. In this context, the approach of linking one single nucleotide polymorphism (SNP) to bevacizumab response may be supplanted by an evaluation of the genetic interaction between SNPs, known as non-linear interaction or epistasis. Moore and collaborators developed and confirmed a procedure named multifactor dimensionality reduction (MDR) analysis to recognize a genetic profile that can predict drug response [43]. Indeed, MDR is a powerful method for finding gene-gene (epistasis) and gene-environment interactions in complex disease studies, especially when traditional logistic regression struggles with high-dimensional data and complex, non-additive effects. To explore this new approach, we conducted two studies on metastatic breast cancer patients [44,45] with the aim of evaluating whether MDR analysis was able to identify favorable pharmacogenetic profiles associated with the best probability of response in terms of efficacy when administered with the antiangiogenic antibody plus first-line paclitaxel. The MDR investigation revealed significant pharmacogenetic interactions, particularly between precise VEGFR-2 rs11133360 and IL-8 rs4073 genotypes, as well as VEGF-A rs833061 and VEGFR-2 rs1870377 genotypes [44,45]. Notably, the favorable profile identified by some VEGF-A rs833061 and VEGFR-2 rs1870377 genotypes was related to both a longer mPFS and mOS,. These encouraging results prompted us to further investigate the capacity of MDR analysis to detect favorable genetic profiles when used in more homogeneous patients’ cohorts, shared by well-known clinical and pathological variables. Exploring this hypothesis, in this latest analysis, we first tested the previously identified favorable genetic profile separating the entire population by hormone receptor expression. The choice was because HR+ tumors identify a different disease compared to those with negative expression of hormone receptors, exhibiting different biological behaviors, targeted treatments, and prognosis.

This allowed us to thoroughly examine how the genetic profile could differently impact patient groups based on their hormone receptor status. Then, we focused on evaluating the MDR analysis on HR+ tumors but introducing a new variable, the time for progression, specifically those patients with a mPFS either greater than 20 months or less than 10 months. This empirical decision-making to exclude patients with a PFS between 10 and 20 months from the analysis, although a harbinger of potentially bias, is based on the hypothesis that the clinical benefit observed in a group of our study population treated with bevacizumab combined with paclitaxel, with a median survival probability of up to 50 months, was due to an underlying genetic profile predictive of response to bevacizumab and the MDR methodology able of identifying it, conscious of the limitations of the MDR methodology itself. However, the advantage of the MDR methodology in identifying multiple genetic profiles potentially capable of predicting a benefit from the combination, as described in our previous works, is offset by the limitation that if the frequencies of these favorable genetic profiles in the study population can be low or may provide a favorable genetic profiles with a significant *p*-value but with a modest impact in terms of PFS and OS, compared to the population with the unfavorable genetic profiles. Another potentially intrinsic limitation of the MDR methodology is due to the sample size required to identify these profiles. So, to overcome limitations above described and to evaluate the potential role of the MDR analysis to identify the pharmacogenetic profile with the highest probability of efficacy in terms of OS, we consciously introduced this empirical choice and performed the analysis in two groups of patients, suggesting that the group of subjects who probably did not take advantage from the introduction of bevacizumab were those with a PFS of less than 10 months, while those with the highest probability of efficacy were patients with a PFS up to 20 months. Finally, patients with an intermediate PFS between 10 and 20 months were excluded from the analysis in order to avoid all those genetic profiles that could be potentially favorable but clinically less relevant. Here, we present the final results of this targeted analysis.

## 2 Patients and Methods

### 2.1 Study Population

The inclusion and exclusion criteria of the original study (clinicaltrial.gov identifier NCT01935102) are described in the article of Coltelli and colleagues [45]. Briefly, inclusion criteria for both groups of patients were the following: age higher than 18 years old; Eastern Cooperative Oncology Group (ECOG) performance status from 0 to 2; hormonal-receptor status; previous adjuvant chemotherapy (none, anthracycline or anthracycline plus taxanes); previous hormonal therapy in adjuvant or metastatic setting; extent of disease (less or more than three sites); location of disease (viscera or bone); measurable or non-measurable disease. Subjects with human epidermal growth factor receptor type 2 (HER2)-positive, were eliminated from the clinical trial. From the initial 307 patients who entered the study [45], the present analysis was restricted to 260 patients with HR+ tumors; then to 168 patients HR+ with a PFS lower than 10 or higher than 20 months: 106 patients administered with the anti-VEGF antibody plus paclitaxel and 62 patients with first-line chemotherapy without the antiangiogenic drug. The first-line treatment consisted of bevacizumab 10 mg/m^2^ intravenously on days 1 and 15 plus paclitaxel 90 mg/m^2^ intravenously on days 1, 8, and 15, every 4 weeks, or chemotherapy without bevacizumab in the control group. Full details are reported in the previous articles [44,45]. Paclitaxel was administered until either disease progression, unacceptable toxicities, or for clinical decision to discontinue. Maintenance with bevacizumab was continued until disease progression, even when paclitaxel was discontinued for other reasons than disease progression. Hormone therapy was introduced at the beginning of bevacizumab maintenance, if not previously used for advanced disease or when progression of disease occurred less than 6 months after the end of adjuvant therapy. Radiological assessment was performed according to the RECIST criteria 1.1 every 8 weeks. In subjects without definable cancer dimensions, disease progression was defined when new lesions appeared, existing lesions evolved, or there was a deterioration of the clinical condition. PFS was defined as the period from the start of treatment to disease progression or death from any reason. OS was described as the time from the start of therapeutic schedule to death from any reason. All subjects were evaluated for PFS and OS. Each patient enrolled in the clinical trial provided informed consent. The study was authorized by the ethics committee of Azienda Ospedaliera-Universitaria Pisana (CESM-AOUP 3077/2010; clinicaltrial.gov identifier NCT01935102) for Massa Carrara, Livorno, Lucca, Pisa, Pontedera and Versilia Hospitals, as well as the ethics committees of all attending structures.

### 2.2 Genotyping Analyses

Blood samples (3 mL) were taken in EDTA vials and kept at −80°C. The genes and polymorphisms implicated in the angiogenic process were picked for analysis on the basis of our prior studies [44,45]. The polymorphisms chosen are listed in supplementary Table 1. Germline DNA extraction was conducted applying the QIAamp DNA Blood Mini Kit (ID #51104; Qiagen, Valencia, CA, USA). Allelic discrimination was performed with an ABI PRISM 7900 SDS system (Applied Biosystems, Carlsbad, CA, USA) employing certified TaqMan^®^ SNP genotyping assays (Applied Biosystems; see supplementary Table 1). PCR reactions were conducted following the manufacturer’s instructions. Genotyping was only carried out once an acceptable number of events (>80% of the enrolled subjects) was reached in terms of progression-free survival (PFS). All samples were genotyped twice to ensure accuracy and reproducibility.

**Table 1:** Characteristics of HR+ metastatic breast cancer patients at baseline treated with paclitaxel (PTX) plus bevacizumab (BEV) or paclitaxel alone.

Characteristics	Subset	PTX+BEV	% PTX+BEV	PTX	% PTX	*p*-Value
Adjuvant CHT	No	45	42.45%	29	46.77%	0.586
	Yes	61	57.55%	33	53.23%	
Adjuvant CHT with taxanes	No	85	80.19%	45	72.58%	0.107
	Yes	21	19.81%	17	27.42%	
Adjuvant HT	No	33	31.13%	18	29.03%	0.775
	Yes	73	68.87%	44	70.97%	
DFI > 12 months	No	32	30.19%	20	32.26%	0.779
	Yes	74	69.81%	42	67.74%	
≥3 Pathological sites	No	67	63.21%	50	80.65%	0.017
	Yes	39	36.79%	12	19.35%	
Visceral disease	No	32	30.19%	25	40.32%	0.180
	Yes	74	69.81%	37	59.68%	
Age (years)	≥65	23	21.70%	28	45.16%	0.001
	<65	83	78.30%	34	54.84%	
Toxicity (grade)	1–2	85	81.00%	54	87.10%	0.304
	3–4	20	19.00%	8	12.90%	
Grading	2	89	84.80%	54	87.10%	0.678
	3	16	15.20%	8	12.90%	

Note: *CHT*, chemotherapy; *DFI*, disease-free interval; *HR*, hormone receptor; *HT*, hormonal therapy.

### 2.3 Statistical Analysis

The analyses were performed as previously described [45]. Briefly, the data analysts in this study were blinded to which samples were sourced from subjects administered with only the anti-tubulin drug vs. those treated with paclitaxel combined with the anti-VEGF antibody. The primary objective of the clinical trial was to recognize a genetic profile favorable in terms of PFS among HR+ MBC subjects administered with a combination of bevacizumab and paclitaxel. OS served as secondary endpoints. The MDR analysis (version 2.0 beta 6, http://sourceforge.net/projects/mdr/, accessed June 2025) was used to explore interactions among gene polymorphisms and recognize genetic profiles linked to improved PFS. The MDR analysis is a non-parametric, model-free strategy for discovering predictive SNP combinations. It pools genotypes from multiple SNPs to create new attributes. Differences in PFS and OS between favorable and unfavorable genetic profiles were evaluated using the log-rank test and the Kaplan-Meier methodology for survival curves. The adjusted HR and 95% confidence interval (95% CI) for potential genetic profiles and medical characteristics were calculated using Cox proportional hazards modeling, with stepwise backward variable selection. Kaplan-Meier and Cox proportional hazards evaluations were executed utilizing SPSS version 17.0 (SPSS, IBM Corp., Armonk, NY, USA). For genotype combinations, the significance of *p*-values was confirmed through 1000-fold permutation testing (please see at https://sourceforge.net/projects/mdr/files/mdrpt/), and the null hypothesis was rejected when the upper-tail Monte Carlo *p* value derived from the permutation test was ≤0.05. As this was an exploratory study without prior published data on the targeted genetic profiles and treatment regimen, power and sample size estimations were not conducted, and no data were excluded from analysis. However, the post-hoc power was calculated. In the treatment paclitaxel alone, the calculated post-hoc power using the observed alpha values was the following: power OS = 0.6041, power PFS = 0.5044. In the treatment paclitaxel in combination with bevacizumab, the calculated post-hoc power with alpha value set as 0.05 was the following power PFS = 0.9089, power OS = 0.9327.

## 3 Results

Initially, the favorable genetic profile previously identified [45] was evaluated separately in hormone-sensitive and triple-negative of the whole 307 breast cancer population. This HR+ population of both cases and control was 260 patients, of which 175 subjects were administered with paclitaxel and bevacizumab, and 85 with only paclitaxel.

In this unselected group of 175 HR+ subjects administered with paclitaxel plus bevacizumab, the mPFS observed was 12.6 (95% CI 11.0–14.1 months) and the mOS was 36.7 months (95% CI, 30.0–43.3 months).

**Figure 1:**
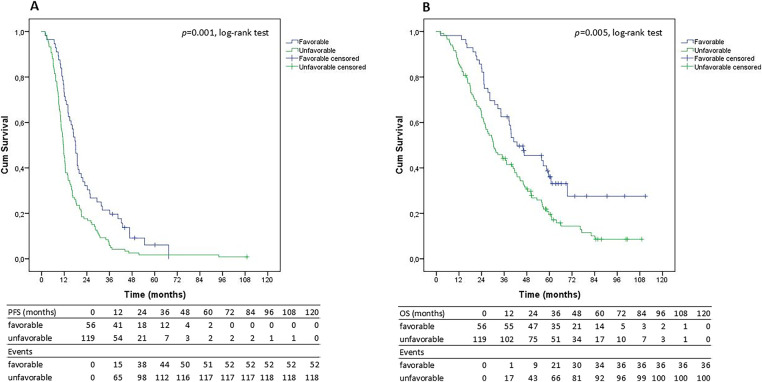
Progression-free survival (PFS; **A**) and Overall survival (OS; **B**) curves in 175 unselected HR+ advanced breast cancer subjects administered with paclitaxel and bevacizumab, analyzed by the Kaplan Meier methodology, according to the favorable (blue line) and unfavorable (green line) genetic profiles. PFS: progression-free survival; OS: overall survival; HR+: hormone receptor-positive.

When the MDR analysis was applied, an improved mPFS in the 56 patients with a favorable genetic profile of 18.1 months (95% CI, 10.6–12.6 months) was observed compared to 11.6 months for the 119 patients with an unfavorable genetic profile (95% CI, 15.8–20.2 months; *p* = 0.001, log-rank test) (Fig. 1A). In the same group of patients, the mOS for patients with the favorable genetic profile was 42.8 months (95% CI, 24.9–60.6 months), compared to 30.4 mOS (95% CI, 24.1–36.6 months) for those with the unfavorable genetic profile (*p* = 0.005, log-rank test) (Fig. 1B). For the 85 patients treated with paclitaxel alone, the mPFS was 10.5 months (95% CI, 8.0–12.9 months), with a mOS of 45.8 months (95% CI, 33.3–58.4 months). The benefit was lost in this group of 85 patients when treated with only paclitaxel, shared both for the genetic profile, favorable vs. unfavorable, respectively (data not shown).

Subsequently, the aim of this last analysis, the MDR analysis, was tested in 168 patients with HR+ tumors and a PFS of less than 10 or more than 20 months: 106 treated with bevacizumab plus paclitaxel and 62 with first-line chemotherapy without the antiangiogenic drug. The key characteristics of patients are presented in Table 1. Despite a significant *p*-value for the age and the number of metastatic sites between the two treatment groups, the inclusion of these possible prognostic factors in the Cox proportional hazards model, used to evaluate the adjusted hazard ratio for PFS and OS, confirmed that the genetic profile was not significant in the control group (data not shown).

The analysis identified two pharmacogenetic profiles regarding precise VEGF-A rs833061 and VEGFR-2 rs1870377 genotypes, linked to higher PFS and OS benefits (favorable), and the other associated with shorter PFS and OS (unfavorable) after the therapeutic approach consisting of paclitaxel combined with bevacizumab (Table 2).

**Table 2:** Genotype combinations of the VEGF rs833061 and VEGFR-2 rs1870377 polymorphisms translated into favorable or unfavorable genetic profiles for PFS and OS. VEGF-A, vascular endothelial growth factor A; VEGFR-2, vascular endothelial growth factor receptor-2.

Favorable Genetic Profiles			
*VEGF-A* rs833061	CT	*VEGFR-2* rs1870377	AT
*VEGF-A* rs833061	CT	*VEGFR-2* rs1870377	AA
*VEGF-A* rs833061	TT	*VEGFR-2* rs1870377	AA
*VEGF-A* rs833061	TT	*VEGFR-2* rs1870377	TT
*VEGF-A* rs833061	CC	*VEGFR-2* rs1870377	TT
**Unfavorable Genetic Profiles**			
*VEGF-A* rs833061	CT	*VEGFR-2* rs1870377	TT
*VEGF-A* rs833061	TT	*VEGFR-2* rs1870377	AT
*VEGF-A* rs833061	CC	*VEGFR-2* rs1870377	AT
*VEGF-A* rs833061	CC	*VEGFR-2* rs1870377	AA

Table 3 shows the characteristics at baseline of subjects administered with the chemotherapeutic drug alone or in combination with the anti-VEGF drug having the pharmacogenetic favorable and unfavorable profiles; no significant differences between the two groups were found. Among the 106 subjects administered with chemotherapy plus bevacizumab, 59 patients had a mPFS equal to or greater than 20 months, and the remaining 47 patients exhibited a mPFS lower than 10 months.

**Table 3:** Characteristics of HR+ subjects administered with paclitaxel combined to bevacizumab with a pharmacogenetic favorable and unfavorable profile at baseline.

Characteristics	Subset	Favorable (N)	%N	Unfavorable (N)	%N	*p*-Value
Adjuvant CHT	No	26	44.1	19	40.4	0.706
	Yes	33	55.9	28	59.6	
Adjuvant CHT with taxanes	No	50	84.7	35	74.5	0.187
	Yes	9	15.3	12	25.5	
Adjuvant HT	No	20	33.9	13	27.7	0.491
	Yes	39	66.1	34	72.3	
DFI ≥ 12 months	No	18	30.5	14	29.8	0.936
	Yes	41	69.5	33	70.2	
≥3 Pathological sites	No	35	59.3	32	68.1	0.353
	Yes	24	40.7	15	31.9	
Visceral disease	No	18	30.5	14	29.8	0.936
	Yes	41	69.5	33	70.2	
ECOG PS	0–1	54	91.5	45	97.8	0.365
	2	5	8.5	1	2.2	
Positive lymph node	No	20	39.2	15	36.6	0.796
	Yes	31	60.8	26	63.4	
Toxicity (grade)	1–2	48	81.4	37	80.4	0.905
	3–4	11	18.6	9	19.6	
Grading	2	48	81.4	41	89.1	0.271
	3	11	18.6	5	10,9	
Age (years)	≥65	48	81.4	35	74.5	0.393
	<65	11	18.6	12	25.5	

Note: *CHT*, chemotherapy; *DFI*, disease free interval; *HR*, hormone receptor; *HT*, hormonal therapy; ECOG. Eastern Cooperative Oncology Group; PS Performance Status.

**Figure 2:**
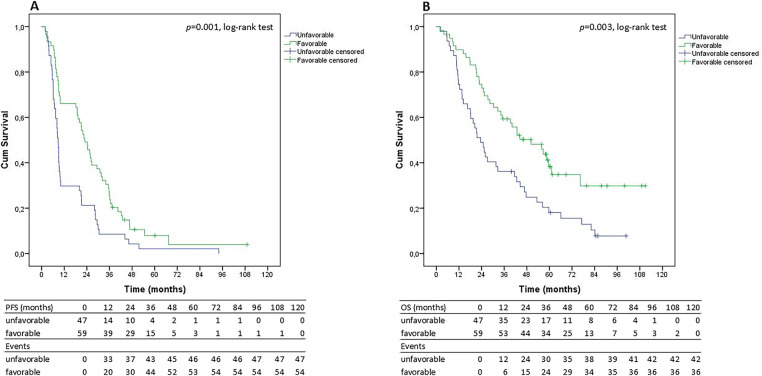
Progression-free survival (PFS; **A**) and Overall survival (OS; **B**) curves in 106 selected HR+ advanced breast cancer patients (with a PFS of less than 10 or more than 20 months) administered with paclitaxel and bevacizumab, calculated by the Kaplan Meier methodology, conforming to the favorable (green line) and unfavorable (blue line) genetic profiles. PFS: progression-free survival; OS: overall survival; HR+: hormone receptor-positive.

In the group of patients treated with bevacizumab and paclitaxel, the mPFS for the favorable genetic profile was 22.9 months (95% CI, 18.1–27.7 months) compared to 8.7 months for the unfavorable genetic profile (95% CI, 7.7–9.7 months; *p* = 0.001, log-rank test; Fig. 2A). The Cox proportional hazards model, utilized to determine the adjusted hazard ratio for PFS in the favorable genetic profile group, displayed a significant result (HR = 0.443, 95% CI 0.284–0.691, *p* < 0.0001; Table 4). The mOS for patients with the favorable genetic profile was 50.2 months (95% CI, 32.2–68.3 months), significantly longer than the 23.5 months (95% CI, 16.7–30.2 months) for those with the unfavorable genetic profile (*p* = 0.003, log-rank test; Fig. 2B). Further analysis using the Cox proportional hazards model, which accounted for other relevant clinical factors, as described in Table 5, revealed that the favorable genetic profile was associated with an adjusted hazard ratio for OS that approached statistical significance (HR = 0.404; 95% CI, 0.249–0.657; *p* < 0.0001). The Cox proportional hazards analysis also evaluated the association between the favorable genetic profile, as well as key clinical characteristics, and both PFS and OS.

**Table 4:** Multivariable Cox regression model of progression-free survival, containing significant variables at the univariate analysis in HR+ subjects administered with paclitaxel and bevacizumab.

Progression-Free Survival (*n* = 106)				
**Characteristics**	**Subset**	**HR**	**95% CI**	***p***
Adjuvant CHT	Yes	1.691	1.072–2.669	0.024
	No	1		
Adjuvant taxanes	Yes	0.424	0.222–0.808	0.009
	No	1		
Sites involvement	≥3	1.710	1.097–2.665	0.018
	<3	1		
Age (years)	≥65	2.370	1.433–3.920	0.001
	<65	1		
Grading	3	0.345	0.184–0.645	0.001
	2	1		
Favorable genetic profile	Yes	0.443	0.284–0.691	<0.0001
	No	1		

Note: *HR*, hazard ratio; *CI*, confidence interval; *n*, number; CHT, chemotherapy.

**Table 5:** Multivariable Cox regression model of overall survival, containing significant variables at the univariate analysis in HR+ subjects administered with paclitaxel and bevacizumab.

Overall Survival (*n* = 106)				
**Characteristics**	**Subset**	**HR**	**95% CI**	***p***
Adjuvant CHT	Yes	1.594	1.594–2.636	0.069
	No	1		
Adjuvant taxanes	Yes	0.371	0.176–0.780	0.009
	No	1		
Sites involvement	≥3	2.118	1.280–3.506	0.004
	<3	1		
Age (years)	≥65	4.628	2.621–8.170	<0.0001
	<65	1		
Grading	3	0.414	0.199–0.864	0.019
	2	1		
Favorable genetic profile	Yes	0.404	0.249–0.657	<0.0001
	No	1		

Note: *HR*, hazard ratio; *CI*, confidence interval; *n*, number; CHT, chemotherapy.

The clinical features assessed included the presence of three or more metastatic sites, prior adjuvant chemotherapy, prior treatment with taxanes, and age <65 or ≥65 years. This comprehensive analysis demonstrated that the favorable genetic profile and the presence of these clinical features were significantly related with both improved PFS and OS (Tables 4 and 5), with the only exception of adjuvant chemotherapy.

Notably, 73.6% of patients with a mPFS of 20 months or more had the favorable genetic profile, while 26.4% had the unfavorable profile. Conversely, in the population with a mPFS of less than 10 months, the favorable profile was observed in only 38% of cases, while the unfavorable profile was present in 62% of patients. Patients treated with chemotherapy alone had a mPFS of 8.7 months (95% CI, 6.7–10.7 months), with no significant difference in outcome based on the presence or absence of the favorable genetic profile: mPFS of 7.4 months (95% CI, 3.8–10.9 months) and 9.2 months (95% CI, 8.1–10.4 months), respectively (*p* = 0.820, log-rank test). Similar results were observed in terms of overall survival (*p* = 0.143, log-rank test). Those with PFS of at least 20 months were comparable to those with PFS less than 10 months in terms of clinical characteristics.

## 4 Discussion

The normal treatment of subjects with MBC HR+ and HER2-negative includes a combination of the CDK 4/6i palbociclib, ribociclib, and abemaciclib, combined with aromatase inhibitors or fulvestrant, and has greatly improved the outcome [4,5].

A recent phase II study in pre-perimenopausal women with clinically aggressive HR+/HER2-tumors has also shown an increase in mPFS for the first-line combination of ribociclib plus aromatase inhibitors and goserelin compared to combination chemotherapy, but without any advantage in objective response rate or even in OS [46]. While a recent meta-analysis revealed that the combination of bevacizumab and paclitaxel was significantly more effective than palbociclib plus letrozole in terms of response rate for postmenopausal patients with HR+ and HER2-metastatic breast cancer [47].

The combinations of CDK 4/6i have extended median survival times to over 50 months during first-line treatment [4,5]. However, disease progression still occurs in nearly half of patients after around 25 months of therapy, and subsequent treatments are needed. In this setting, according to the patient’s clinical condition and extension of disease, further endocrine therapies (ET) can be proposed, but none are widely accepted as the standard treatment.

Novel Akt inhibitors, such as capivasertib [23] or the oral selective estrogen receptor degraders [11,21,22], are valid options, but over time, progressed disease occurred, and chemotherapy remains the forced choice. Trastuzumab deruxtecan [48] and sacituzumab govitecan [49] represent an option in the HR+/HER2-low breast cancer population but have been approved by the FDA and EMA only after a first-line chemotherapy [50,51].

Thus, despite these advancements, chemotherapy remains an unavoidable option in the whole group of advanced breast cancer HR+/HER2-patients, regardless of both ET and targeted therapies previously undertaken. Therefore, bevacizumab combined with paclitaxel could be a valid choice in this setting, especially when predictive factors of response can lead towards this therapeutic choice.

The MDR analysis was previously investigated to identify genetic polymorphism interaction profiles that could predict toxicity of cancer drugs such as methotrexate [52] and both therapeutic response in metastatic colorectal cancer [53] or breast cancer patients [44,45]. Indeed, the complexity of the therapeutic schedules and the frequent combination of various antineoplastic drugs suggest a change of experimental approach from single nucleotide polymorphisms (SNPs) to a more composite gene interaction investigation to anticipate the efficacy of antineoplastic drugs. Sample size necessities for MDR are not yet known, and for accurate MDR modeling involving multiple SNPs sample size of 168 could be small. A total sample dimension of 400 patients has been demonstrated to have fine power to discover two-locus interactions for a precise set of epistasis models played in datasets of ten total SNPs [54].

The MDR analysis was applied in our previous analyses to assess the impact of combining bevacizumab and paclitaxel vs. paclitaxel alone in MBC patients with different genetic profiles. The aim was to explore the potential to identify the most favorable genetic profile for predicting PFS and OS. The MDR investigation revealed relevant pharmacogenetic interactions, particularly between precise VEGFR-2 rs11133360 and IL-8 rs4073 genotypes, as well as VEGF-A rs833061 and VEGFR-2 rs1870377 genotypes [44,45]. Notably, the favorable profile identified by VEGF-A rs833061 and VEGFR-2 rs1870377 genotypes was related with both a longer median PFS (16.8 vs. 10.6 months, *p* = 0.0011) and median OS (39.6 vs. 28 months, *p* = 0.011) compared to the unfavorable genetic profile, as described previously [45]. The encouraging results obtained with the MDR analysis prompted us to further investigate the possibility of identifying subgroups of patients most likely to respond to the combination treatment.

Firstly, we tested the previously identified favorable genetic profile on the entire study patient population, separating them by hormone receptor expression. The favorable profile showed a significant benefit in the HR+ population, with improved mPFS and mOS, but not in HR- tumors. These results confirmed that HR+ and HR- tumors have different biology and evolution [55,56], suggesting the hypothesis that the MDR analysis should be applied separately for the two subgroups of patients to identify a favorable genetic profile. Therefore, we continued the analysis only in patients whose tumors expressed hormone receptors, further selecting them based on PFS.

This allowed for a more focused analysis, minimizing the confounding effects of variability typically seen in these broader patient populations. Thus, applying the MDR analysis only on the basis of hormone receptor expression and introducing an additional variable such as mPFS duration greater than 20 months or less than 10 months, the mOS increased dramatically up to 50 months for those patients with a favorable genetic profile. Notably, in more than 80% of patients, a progression of disease occurred during first-line hormone therapy, including an aromatase inhibitor. At the time of the study, CDK 4/6i were not available in this setting of patients. Furthermore, as described, more than 70% of patients with a mPFS longer than 22 months exhibited this favorable genetic profile.

Therefore, these data suggest that the MDR analysis can help us to identify the patients most likely to benefit from bevacizumab plus paclitaxel, but that additional pathological or clinical variables could enhance the ability of MDR to identify patients with an even higher probability of response.

In the context of a personalized medicine approach, if these preliminary findings are subsequently confirmed in further studies, a test that predicts the best response to treatment with bevacizumab and paclitaxel could assist in selecting the appropriate therapy for patients with advanced HR+ breast cancer, progressed to first-line cyclin-based therapies or, in any case, when chemotherapy is indicated independently of previous treatments.

In our study, we used MDR analysis to show a statistical interaction between SNPs of the VEGF-A and VEGFR-2 genes, which might affect the efficacy of bevacizumab on both PFS and OS. Due to the interconnected nature of biological networks, a single polymorphism may influence thousands of gene-gene interactions, leading to a variety of phenotypes. Gene-gene interactions are recognized as crucial components of gene modulation, signal transduction, and biochemical and physiological processes leading to the event called biological epistasis [57]. Assessing the link between biological and statistical epistasis [58] may be complex because the first one occurs at the individual level, whereas the second one is observed at the population level and arises from interindividual differences in genotypes, biomolecules, and their interactions, such as how bevacizumab is effective in advanced HR+ breast cancer patients. It is evident that formulating hypotheses or drawing deductions regarding biological functions and causation from statistical data is challenging, particularly if essential biomolecular data has not been investigated. The two genes and the proteins they encode are part of the same signaling pathway, and it’s well-established that VEGF-A promotes VEGFR-2 phosphorylation and cancer-related angiogenic process [59]. Given this context, it can be hypothesized and speculated that in patients with a favorable genetic profile, the HR+ cancer cell mediated angiogenesis is effectively inhibited when bevacizumab is present. The effectiveness of bevacizumab in blocking angiogenesis may be due to higher levels of VEGF-A production associated with the VEGF-A rs833061 TT genotype or T allele, as the T allele of the SNP VEGF rs833061 C > T has been linked to increased promoter activity [60], possibly leading to a more dependent tumor angiogenesis to VEGF-A stimulus and, thus, to its pharmacological block. Additionally, the VEGFR-2 rs1870377 SNP, which features a glycine to histidine substitution (Q472H) in the receptor’s extracellular ligand-binding domain [61], might influence VEGFR-2 degradation. In a favorable genetic profile, the VEGFR-2 rs1870377 AA genotype or A allele synthesizes a receptor that is structurally changed, suggesting it is extremely activated or reduced, less responding to the remaining free-VEGF-A after bevacizumab treatment. Conversely, in subjects with an unfavorable genetic background, different genotype combinations may lead to decreased VEGF-A production and thus to a less dependent tumor from VEGF-A induced angiogenesis or normal VEGFR-2 presence on tumor endothelial cells, potentially allowing their proliferation, migration, or survival since bevacizumab does not fully block VEGF-A action. This suggested mechanism is a speculative hypothesis to work on because of the lack of molecular data from patient samples, and only future clinical trials may confirm this possible link between favorable profile and phenotypes.

So, the findings from these analyses, conducted in an unselected group of patients, raise reasonable questions about what next steps should be taken. Potentially, more patients are selected on the basis of clinical or pathological variables, and more accurately, the MDR analysis identifies who could benefit most from the treatment, though with a favorable genetic profile. The main drawback of our study was the sample size, which did not allow us the possibility to explore the role of other variables.

The results of these last analyses could suggest the presence of a favorable genetic profile potentially capable of selecting patients with the highest probability of response in terms of survival when treated with bevacizumab plus paclitaxel in a population of patients predominantly with disease progression after a first-line hormonal therapy based on aromatase inhibitors, and more generally to the hormone-resistant population. These observations remain speculative due to the limitations outlined in the introduction and related to the conduct of the study such as the ambidirectional nature of the trial, the lack of blinding, the possible small sample size required for MDR analysis, the absence of patients in the study who had received CDK 4/6i, and the empirical choice to exclude patients with a PFS between 10 and 20 months from the analysis. These issues represent biases that do not allow conclusions to be drawn that would change clinical practice. Moreover, due to the small control group treated with paclitaxel alone, also the interpretation of the role of genetic profiles in this setting of patients needs a considerable degree of caution. However, they could contribute to an important clinical question. Namely, what could be the best choice of chemotherapy when this remains the only reasonable option for patients diagnosed with advanced HR+ breast cancer, once all hormonal manipulations have been exhausted after a first-line treatment that includes an aromatase inhibitor and a CDK 4/6i, the current standard of care in this setting of patients. Although chemotherapy remains the only option in resistant HR+ tumor, it is now relegated to a marginal role with a sequential strategy as the only alternative. Maintaining treatment with a low probability of toxicity remains the main objective, with the expectation that it will guarantee adequate efficacy. In this context, exploring new approaches to enhance the effect of chemotherapy remains mandatory, and these results, although speculative, could therefore provide the rationale for designing a prospective multicenter blinded study, the only way to validate our initial hypothesis of tailored therapy with bevacizumab combined with paclitaxel.

Furthermore, it is important in future trials not only to use MDR analysis but also other possible blood-based biomarkers such as the exosomal miRNAs due to their high stability, secretion from malignant cells, and high selectivity for different breast cancer subtypes [62].

In summary, the genetic analysis of the interaction between single-nucleotide polymorphisms may reveal promising pharmacogenetic biomarkers for bevacizumab-combined therapies, rather than the examination of a unique SNP in a sole gene. The MDR analysis can be effectively utilized for this purpose, as evidenced by the findings in this metastatic HR+ breast cancer patient population, where the study of the interaction between VEGF-A rs833061 and VEGFR-2 rs1870377 gene polymorphisms led to the documentation of a genetic profile related to prolonged PFS and OS.

## Data Availability

The data that support the findings of this study are available from the corresponding authors, upon reasonable request.
